# Exercise and Adipose Tissue Macrophages: New Frontiers in Obesity Research?

**DOI:** 10.3389/fendo.2016.00065

**Published:** 2016-06-14

**Authors:** Jorming Goh, Kian Peng Goh, Asghar Abbasi

**Affiliations:** ^1^Combat Protection and Performance Program, DSO National Laboratories, Defence Medical and Environmental Research Institute, Singapore; ^2^Department of Physiology, Yong Loo Lin School of Medicine, National University of Singapore, Singapore; ^3^Division of Endocrinology, Department of Medicine, Khoo Teck Puat Hospital, Singapore; ^4^Institute for Memory Impairments and Neurological Disorders (MIND Institute), University of California Irvine, Irvine, CA, USA

**Keywords:** obesity, exercise, adipose tissue, macrophages, inflammation, polarization

## Abstract

Obesity is a major public health problem in the twenty-first century. Mutations in genes that regulate substrate metabolism, subsequent dysfunction in their protein products, and other factors, such as increased adipose tissue inflammation, are some underlying etiologies of this disease. Increased inflammation in the adipose tissue microenvironment is partly mediated by the presence of cells from the innate and adaptive immune system. A subset of the innate immune population in adipose tissue include macrophages, termed adipose tissue macrophages (ATMs), which are central players in adipose tissue inflammation. Being extremely plastic, their responses to diverse molecular signals in the microenvironment dictate their identity and functional properties, where they become either pro-inflammatory (M1) or anti-inflammatory (M2). Endurance exercise training exerts global anti-inflammatory responses in multiple organs, including skeletal muscle, liver, and adipose tissue. The purpose of this review is to discuss the different mechanisms that drive ATM-mediated inflammation in obesity and present current evidence of how exercise training, specifically endurance exercise training, modulates the polarization of ATMs from an M1 to an M2 anti-inflammatory phenotype.

## Introduction

The immune system is instrumental in mediating a number of physiological processes in the mammalian species, including pathogen surveillance, wound repair, and metabolic regulation. Accumulating evidence shows that the immune system interacts with other organ systems, including the adipose tissue. Traditionally, adipose tissue is known for its role in energy homeostasis, especially as a storage depot for lipids. The scientific paradigm of this once-neglected organ shifted, when researchers discovered novel secretory functions of adipose tissue in the mid-1990s. Seminal studies demonstrated that gene and protein products of hormones that regulate satiety, such as leptin, were found to be overexpressed in, and secreted from adipocytes (1, 2).

Adipose tissue was further recognized as an endocrine-like organ, particularly after adipose tissue *per se* was shown to express and secrete cytokines that can exert effects in distant organs, such as tumor necrosis factor (TNF)-α and interleukin (IL)-6, among other cytokines previously thought to originate only from immune cells (3, 4). Cytokines, hormones, and other protein factors secreted from adipose tissue were subsequently termed “adipokines,” and they can exhibit autocrine, paracrine, and endocrine functions (5). Since then, adipose tissue has also been recognized to be in a chronic inflammatory state in an obese host, wherein immune cells, such as macrophages, were found in greater abundance – 40% of all cells within white adipose tissue (WAT) of obese mice, relative to 10% in lean mice (6). In addition, this increased macrophage density correlated with glucose and insulin resistance (7). In an obese state, more than 90% of all adipose tissue macrophages (ATMs) were found at sites of adipocyte death, where they form a crown-like structure (8) and participate in tissue remodeling, such as scavenging lipids from necrotic adipocytes (9) or inducing vessel growth (10).

Resident macrophages are phenotypically heterogeneous, where two distinct forms of macrophages, M1 and M2 phenotypes, are found within adipose tissue of both obese and lean individuals (or mice), with the extent of each phenotype dependent on local signals from the adipose microenvironment. In general, murine studies have demonstrated that excess adiposity increases the proportion of M1 to M2 macrophages in WAT (8). Lumeng’s group reported that after diet-induced obesity, murine ATMs had high gene expressions of cluster of differentiation (CD)11c^+^, TNF-α, and inducible nitric oxide synthase (iNOS), which are markers characteristic of M1 macrophages, whereas ATMs from lean mice expressed many genes characteristic of M2 macrophages, including arginase 1 (Arg1), Ym1, and IL-10 (8).

## A Caveat on M1/M2 Macrophage Phenotypes

It is important to note that the M1/M2 nomenclature is an oversimplification of the polarized macrophage phenotype, with other subsets, such as M2a that can also be found in adipose tissue, although the proportions would vary based on physiological conditions. For example, the ratio of M2a to M1 murine ATMs in high-fat diet-induced obesity was approximately 1.2:1, but in lean mice it was 4:1 (11). Furthermore, although CD11c^+^ M1 macrophages accumulate after 8 weeks in adipose tissue of high-fat diet fed mice, these macrophages also presented with increased M2 macrophage gene expression (Arg1, IL-1ra) as well as other genes related to matrix remodeling (12). It is interesting to note that after 12 weeks of the high-fat diet, the ATMs presented with decreased M1 macrophage gene transcripts (e.g., IL-1β) and upregulated M2 macrophage gene transcripts (e.g., Ym1). Moreover, genes involved in oxidative metabolism, such as peroxisome proliferator-activated receptor (PPAR) gamma co-activator 1-alpha (PGC-1α) were found to be upregulated (12). Hence, the polarized state of ATMs is not constant, but in dynamic flux. Studies that have single time-point measurements of ATMs may, thus, miss the dynamic changes in M1 and M2 populations during obesity and exercise intervention. In addition, a recent study by Kratz’s group (13) demonstrated that polarization of ATMs by metabolic substrates, such as palmitate and glucose *in vitro* induced a distinct form of macrophage, which did not express surface markers coincident with classical activation, but rather, upregulated proteins involved in lipid metabolism, such as CD36, ATP-binding cassette transporter (ABCA)-1 and Perilipin-2, which are associated with M2 macrophages. The data from this group further suggest that the M1 and M2 paradigm is more complex, and in metabolic disease, may need further elucidation of the polarized phenotypes to better distinguish from other disease models.

In view of the diverse phenotypes reported in the literature, we would like to recommend a standardized experimental approach to harmonize future research findings in this area. At the International Congress of Immunology in Milan in 2013, a group of macrophage biologists met to discuss the current limitations in the literature pertaining to the activation or polarization of macrophages (14). The major challenge with the macrophage nomenclature stems from the use of terminology, where in addition to M1 and M2, other terms such as “classical” and “alternative,” “regulatory” and subsets of M1 and M2 have been described. Such nomenclatures resulted from disparate experimental approaches, for instance, whether macrophages were activated *in vitro* by IL-4 or IFN-γ, which gave rise to “alternative” and “classical” macrophage phenotypes, respectively. Alternatively, M1 or M2 classification has also been reported, depending on the growth factors used, for instance, granulocyte macrophage colony-stimulating factor (GM-CSF) or CSF-1 have been used to denote M1 and M2 macrophages cultured *in vitro* with these growth factors, respectively. Hence, at the Milan congress, the macrophage biologists came up with a framework for studies reporting macrophage phenotypes. The framework essentially calls for investigators to be more precise in (i) describing the source of macrophages (bone marrow, peritoneal, or peripheral macrophages), (ii) specifying the conditions in which macrophages were activated, and (iii) to use a nomenclature consistent with the spectrum of activation states (14).

In this review, we will use the generic nomenclature to denote M1 vs. M2 macrophage phenotypes, with the premise that an M1 phenotype is pro-inflammatory in that these macrophages produce cytokines, such as TNF-α, IL-6, and IL-12, whereas a M2 phenotype is anti-inflammatory, and these M2 macrophages produce large amounts of anti-inflammatory cytokines, such as IL-10 and IL-1 decoy receptor (8). In the next few sections, we will discuss how the excess adiposity contributes to molecular changes in the adipose tissue, leading to the activation and polarization of ATMs, and how regular exercise may reverse the macrophage phenotypes seen in obesity.

## Obesity-Related Metabolic Stress, Inflammation, and Macrophage Polarization

Obesity- and exercise-induced macrophage polarization requires an integration of metabolic and immune crosstalk. First, excess adipocyte lipid availability during obesity increases (i) the availability of fatty acids for activation of immune and metabolic mediators of inflammatory response, including toll-like receptor (TLR)-4, nuclear factor-kappa B (NF-κB), IkB kinase (IKK)-β, Jun kinase (JNK)-1, fatty acid binding proteins (FABPs), PPAR(s), (ii) cellular stress [unfolded protein response (UPR) in the endoplasmic reticulum], (iii) mitochondrial reactive oxygen species (ROS) production and mitochondrial dysfunction, and (iv) protein synthesis (mammalian target of rapamycin (mTOR) hyperactivation) (15–17). Each of these factors will be discussed in turn.

### Excess Availability of Fatty Acids

In the obese state, increased concentrations of saturated fatty acids activate (i) TLR4 in both adipocytes and macrophages (18), (ii) NF-κB in adipocytes (19), (iii) IKKβ in myeloid cells (20), and (iv) JNK in adipocytes (21), all of which activate downstream inflammatory cytokines and proteins, such as TNF-α, IL-6, and iNOS (16). These pro-inflammatory cytokines and proteins participate in a feedback loop between adipocytes and circulating monocytes, culminating in the M1 polarization of macrophages that infiltrate the adipose tissue.

Although well characterized as a cellular lipid chaperone, ligand-bound FABP4 also demonstrates novel roles in inflammation and in its interaction with nuclear receptors, such as PPARs (15). FABP4^−/−^ macrophages demonstrated impaired IKK and NF-κB activity, concomitant with a reduction in protein expressions of cyclooxygenase (COX)-2 and iNOS, as well as lower LPS-stimulated secretions of monocyte chemotactic protein (MCP)-1, TNF-α, and IL-6 (22). Obesity-associated hyperlipidemia presents excess fatty acids that bind to FABP4 in adipocytes or stromal macrophages in the adipose tissue microenvironment, inducing the secretion of pro-inflammatory cytokines that recruit greater numbers of M1 macrophages. This view is supported by co-culture experiments where deletion of FABP4 in adipocytes resulted in MCP-1 gene expression in macrophage, and deletion of FABP4 in macrophages improved insulin signaling and glucose uptake in adipocytes (23).

Peroxisome proliferator-activated receptors are transcription factors that can be activated by fatty acids, and belong to the nuclear receptor family (24). They are expressed in adipose tissue, skeletal muscle, liver, macrophages, and other organs, although the three isoforms (α, δ, and γ) are tissue specific (25). Both PPARγ and PPARδ expressions are negatively associated with obesity, where they modulate adipogenesis and lipid oxidation, respectively (26). In addition, PPARδ is required for inducing macrophage M2 polarization, as PPARδ^−/−^ macrophages demonstrated a decrease in M2 macrophage profile expression, as gene expressions of IL-13, IL-4, and macrophage galactose *N*-acetyl-galactosamine were all reduced, compared with wild-type macrophages (27). In addition, myeloid-specific PPARδ^−/−^ mice demonstrated an increase in gene expression of M1 macrophage markers (MCP-1, TNF-α, IL-6) (27). Similarly, PPARγ^−/−^ macrophages are polarized toward the M1 phenotype (28), suggesting that PPARs play a role in mediating macrophage polarization.

### Mitochondrial ROS, Cellular Stress, and Aberrant Protein Synthesis

Obesity augments metabolic stress in the organism. At the cellular level, this is demonstrated through mitochondrial dysfunction and mTOR hyperactivation, whereby the integrated cellular signaling of these two pathological conditions can contribute to ER stress (15). Excess lipids in the adipocyte increase the substrate load for inefficient mitochondrial oxidative phosphorylation, leading to generation of ROS that can damage mitochondrial constituents, and perpetuate a cycle of ROS-induced damage and further mitochondrial dysfunction. Adipocyte mitochondrial dysfunction permits further excess cellular lipid build-up, leading to the production of the pro-inflammatory cytokines described earlier and recruitment and polarization of M1 macrophages. Overabundance of lipids in the adipocyte is sensed by mTOR as a high intracellular energy state, leading to hyperactivation of mTOR, which chronically can lead to uncontrolled protein synthesis, increased ER stress, UPR, and JNK activation (15). Lipid overload can also directly mediate the inflammatory response by inducing ER stress, *via* the initiation of UPR (15). The UPR activates cyclic-AMP-responsive element-binding protein H (CREBH), which induces C-reactive protein (CRP) and serum amyloid P-component (SAP) production, both of which are mediators of the acute phase response that contributes to pro-inflammatory cytokine production.

Lipid overload can indirectly simulate the pro-inflammation state, as the ER responds to both mitochondrial dysfunction and mTOR hyperactivation and integrates their respective signals to generate ROS (15) and activate the JNK- (29) and NF-κB-mediated (30) inflammation pathways. Thus, the ER, mTOR, and mitochondria can both directly and indirectly induce M1 macrophage activation *via* production of pro-inflammatory cytokines directly, and through inter-organelle crosstalk as described.

In addition to lipid overload, glucose intolerance and insulin resistance are associated with obesity, which may be partly explained by reduced serum concentrations of adiponectin (31), an important adipokine that promotes glucose uptake, insulin sensitivity, and β-oxidation in peripheral tissues. Adiponectin is an anti-inflammatory and M2 macrophage polarizing molecule, as it attenuates TLR4-mediated NF-κB activation (32) and upregulates IL-10 production in macrophages (33). In addition, adenoviral delivery of adiponectin in wild-type mice increased Arg1 expression in peritoneal macrophages, whereas peritoneal macrophages isolated from adiponectin^−/−^ mice showed increased M1 macrophage polarization (34). Thus, adiponectin deficiency in obesity could alter macrophage polarization fates to favor M1 activation.

## Effects of Exercise on Metabolic Stress, Inflammation, and Macrophage Polarization

### Attenuation of Metabolic Stress

Exercise training increases adipocyte-specific gene and protein expression of AMPK and PGC-1α (35), which in turn enhances β-oxidation and mitochondrial biogenesis, allowing for greater lipid oxidation per mitochondrion. Improved mitochondrial β-oxidation reduces oxidative stress and mitochondrial dysfunction, thus limiting pro-inflammatory cytokine production and reducing signals for ER stress-mediated inflammation. Improved function and reduced stress in both organelles as direct or indirect consequences of exercise training can, thus, attenuate macrophage M1 polarization or recruitment *via* reduced stress signaling. Improved exercise-induced lipid oxidation attenuates the need to transport excess fatty acids. For instance, FABP4 concentrations in circulation were reduced with aerobic training in obese women (36). This attenuation in serum FABP4 may be mediated by improved AMPK signaling, since metformin, an AMPK agonist, reduced macrophage Forkhead box O1 (FOXO1)-mediated transcription of FABP4 protein expression (37).

Peroxisome proliferator-activated receptors also respond favorably to exercise training in that protein expression of PPARδ increased by 53% in adipose tissue of exercise-trained rats fed a high-fat diet, compared with sedentary rats on the same diet (38). Similar outcomes in PPARγ were attained with (i) exercise-trained rats, with increased DNA-binding activity in adipocytes (39) and in (ii) exercise-trained humans, with increased gene expression in adipocytes (35). Such outcomes translate to improved adipocyte lipogenesis and oxidation, reducing free fatty acids in the adipose tissue microenvironment for PPARδ/γ-mediated M1 macrophage activation.

Exercise training improves glucose and insulin sensitivity, mediated partly by improved AMPK/insulin receptor substrate (IRS)-1/phosphoinositol-3 kinase (PI3K) signaling. Such exercise-induced improvements are associated with increased gene (40–42) and protein (43) expression of adiponectin and gene expression of adiponectin receptor (40) in adipose tissues of rats and humans.

### Reduction of Inflammation and Modulating Phenotypes of Macrophages

Physical inactivity has been associated with several chronic metabolic and inflammatory diseases, such as type 2 diabetes mellitus (T2D) (44–46). Furthermore, a sedentary lifestyle is accompanied by the accumulation of visceral fat, which predisposes adipose tissue to infiltration by pro-inflammatory immune cells, increases adipokine secretion and the development of a low-grade, systemic inflammatory state (47). Low-grade systemic inflammation is associated with the pathology of several diseases, including neurodegenerative diseases and insulin resistance. Chronic moderate exercise, in contrast, has been shown to exert anti-inflammatory effects and, therefore, protects against chronic inflammation-associated diseases (44–46, 48). This protective effect of regular exercise may be mediated through both the reduction of visceral fat mass and the induction of an anti-inflammatory environment with each bout of exercise (48, 49).

Several possible mechanisms have been described regarding the beneficial anti-inflammatory effects of regular physical activity, including: (i) reduction in visceral fat mass (with a subsequent decreased production and release of pro-inflammatory adipokines), (ii) reduction in the expression of TLRs on monocyte and macrophages (50), and (iii) induction of several anti-inflammatory molecules from leukocytes and skeletal muscle (51, 52). In addition, the inhibition of monocyte/macrophage infiltration into adipose tissue and the phenotypic switching of macrophages within adipose tissue (52, 53) have been proposed recently. The last two mechanisms are of great importance, since obesity is accompanied by ATMs infiltration into adipose tissue, and induces a phenotypic switch in ATM polarization from an anti-inflammatory M2 phenotype to a pro-inflammatory M1 phenotype and, hence, contributing to insulin resistance (27, 54–56).

The anti-inflammatory function of exercise might prevent chronic inflammatory diseases through the induction of phenotypic switching from M1 to M2 macrophages, as well as inhibit macrophage infiltration into adipose tissue. There are few studies investigating the role of exercise training on macrophage phenotype switching in adipose tissues. In an earlier study by Kawanishi and colleagues (53), these investigators showed, for the first time, that treadmill running (16 weeks) significantly decreased CD11c (M1 macrophage-specific marker) mRNA expression and increased CD163 (M2 macrophage marker) mRNA expression in adipose tissues of obese mice. In a recent study, these authors showed that exercise training decreased TNF-α mRNA and CD11c levels in the adipose tissues of high-fat-diet obese mice (57).

Similar observations were also reported in other pre-clinical studies (58, 59). First, Oliveira et al. (58) reported that two single bouts of swim exercise of 3 h each and separated by 45 min of rest, induced a M1-to-M2 phenotype switch in WAT and stromal vascular fraction (SVF) of rats on a high-fat diet, as evidenced by the increased protein expression of macrophage galactose-type C-type lectin1 (MGL1), a M2 macrophage marker. By contrast, protein expression of TNF-α and iNOS were downregulated in the SVF. Likewise, chronic treadmill running (up to 12 weeks) resulted in the attenuation of CD11c in the adipose tissue of mice on high-fat diets, although surprisingly, the gene expression of two M2-macrophage markers, Arg1 and CD206, was increased in sedentary mice on the high-fat diet, and decreased in chronically trained mice, also on the high-fat diet (59) The authors suggest that the improvements in inflammatory profiles in these mice may involve an attenuation of both M1 and M2 macrophages in adipose tissue.

In humans, only a single study (60) to date had investigated outcomes involving macrophage polarization after exercise training. In this randomized controlled trial that spanned 12 weeks, overweight (BMI: 25–30 kg/m^2^, body fat >25%), young men were stratified into one of four experimental groups: (i) endurance training group, (ii) dietary control group, (iii) endurance training and increased diet without weight loss group, or (iv) control group. Protein expression of CD68, a pan-macrophage marker, was not significantly different among the four conditions; however, CD163, a M2 macrophage marker was increased in subcutaneous adipose tissue of both exercise groups, but not in either the dietary control group or the control group.

Given the paucity of studies that investigated exercise and macrophage phenotype switching, it is clear that more research is needed to determine the proximal signaling pathways that guide M1 vs. M2 macrophage modification in adipose tissues by exercise. It is possible that exercise may indirectly regulate macrophage phenotype through the enhancement of blood-derived or contracting muscles-derived M2-type marker production. We and others have clearly shown that exercise strongly induced the expression and release of IL-10, Arginase-1, CD163, and IL-6 from human leukocytes and skeletal muscles (49–51).

The mechanism by which exercise induces macrophages polarization toward an M2 phenotype is likely to be related to the induction of PPARγ and its co-factors (PGC-1α/β). PPARγ and PGC-1a are known for their important roles in the regulation of efficient energy utilization and oxidative phosphorylation, both of which are reduced in obesity and insulin resistance (61, 62). In particular, PPARγ plays an important role in controlling adipose tissue inflammation and insulin resistance through the activation and infiltration of alternatively activated (M2) macrophages (63, 64). A previous study by Odegaard and colleagues (28) showed that PPARγ deficiency in macrophages impairs M2 macrophage activation and predisposes the animals to development of diet-induced obesity, insulin resistance, and glucose intolerance. Further evidence for the role of PPAR-γ in macrophage activation stems from studies by Stienstra et al. (64) who showed a repolarization of adipose tissues macrophages to an M2 phenotype following treatment of mice with PPARγ agonist. The authors speculated that M2 macrophages might play a role in PPARγ-dependent expansion and remodeling of adipose tissue.

**Figure 1:**
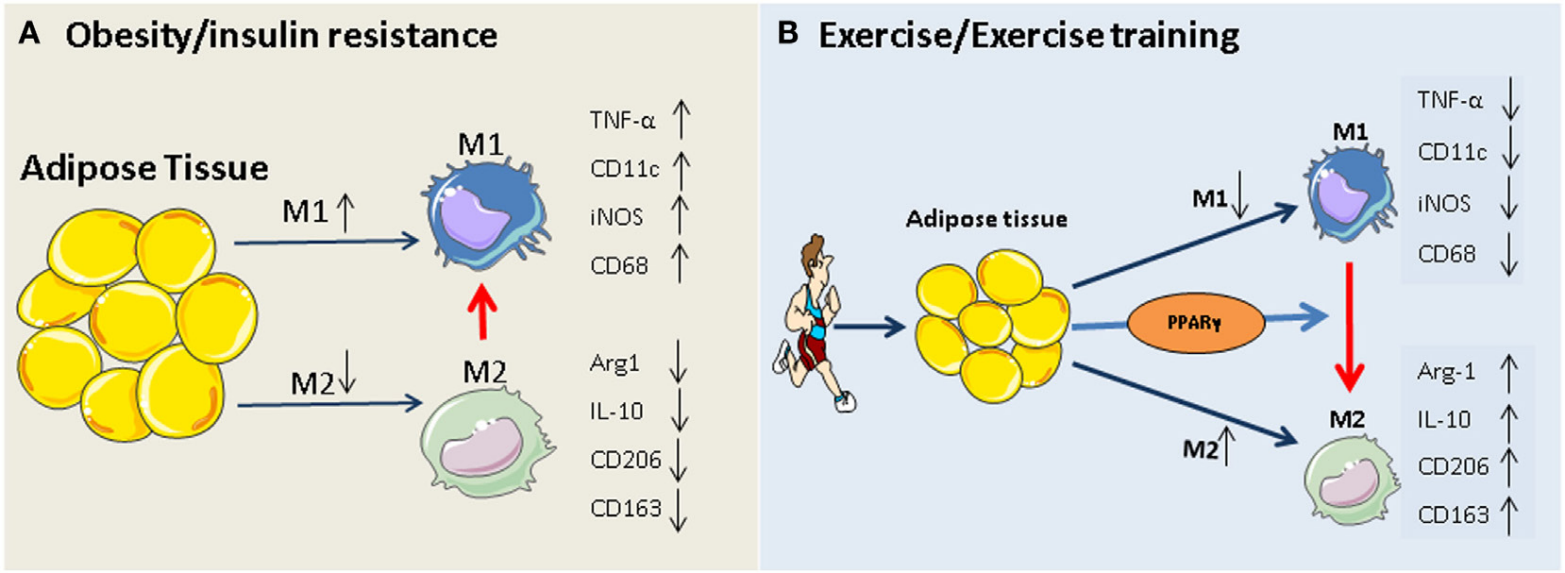
**Potential mechanism of exercise-induced anti-inflammatory function in adipose tissues**. **(A)** Obesity and insulin resistance induces a phenotypic switch in adipose tissue macrophage polarization from M2 type to M1 type macrophages. This is accompanied by the induction of M1-specific markers (such as TNF-α and iNOS) and reduction of M2 markers (IL-10, arginase-1). **(B)** Regular exercise may induce a switch from an M1 to an M2 macrophage phenotype. This, in turn, may contribute to a reduction in the release of pro-inflammatory cytokines (such as IL-6 and TNF) and an increase in the release of anti-inflammatory cytokines (such as IL-10, arginase-1, and adiponectin) from adipose tissue. The exercise-induced phenotypic switch in macrophage type might be mediated by activation of PPARγ.

The PPARγ hypothesis becomes more convincing, given that participation in exercise programs activates PPARγ and PPARγ-mediated signaling events in adipose tissue and monocytes/macrophages (35, 65–68), and that, exercise-induced activation of M2 macrophages is mediated via PPARγ and its co-factors (PGC-1α/β) (69). The beneficial role of exercise-induced PPARγ in serum lipid profiles (increased HDL-cholesterol and decreased total cholesterol, LDL-cholesterol, and triglycerides) has also been reported previously (67, 70). Thus, exercise-triggered adipocyte- and/or monocyte/macrophage-specific PPARγ activation may constitute an additional rationale for prescribing exercise in obesity and type 2 diabetes. Our working hypothesis of how exercise modulates macrophage polarization is summarized in Figure 1.

## Summary

**Figure 2:**
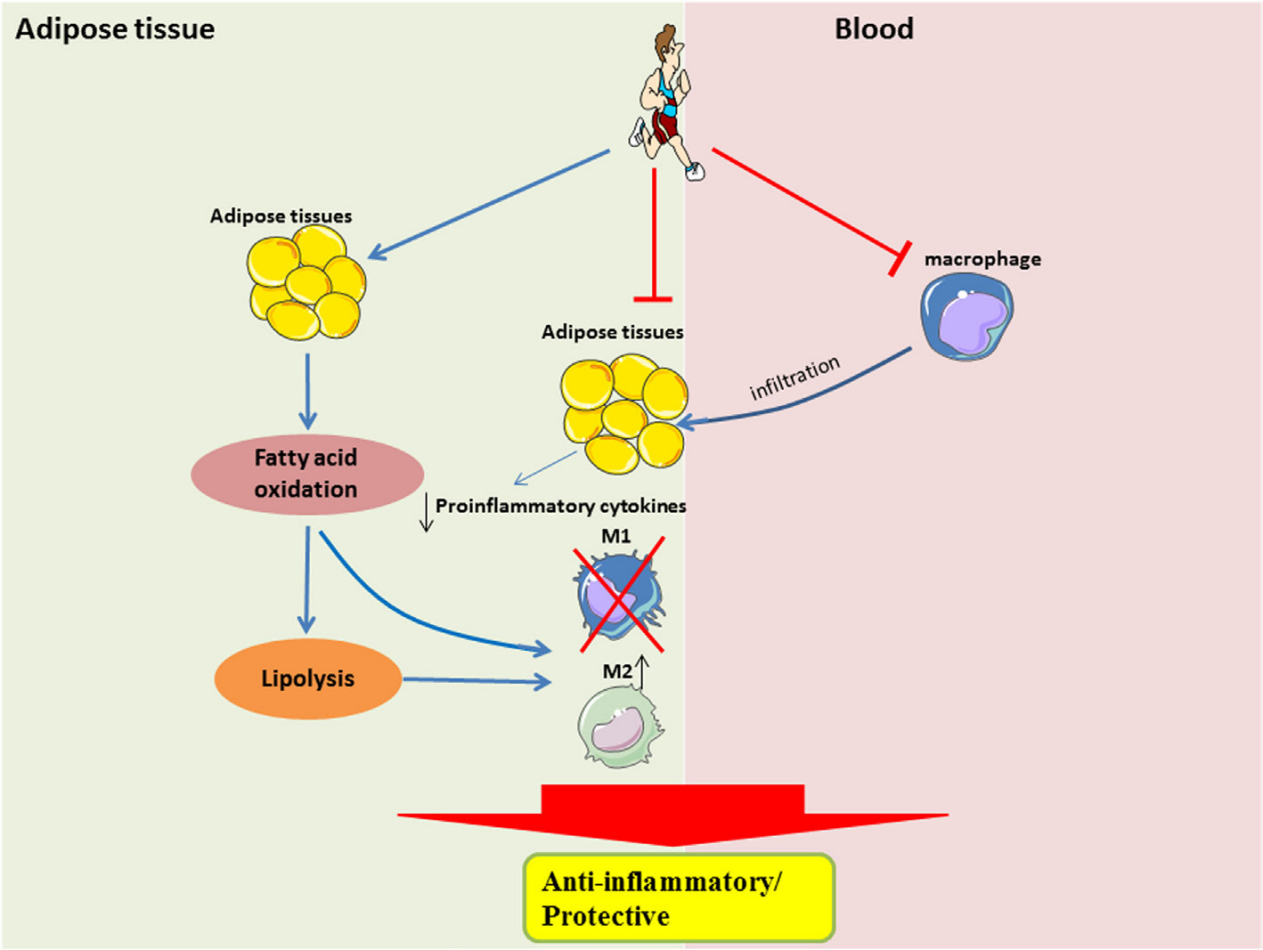
**Systemic and local role of exercise training in modulating substrate utilization and macrophage polarization in adipose tissue**. In the local adipose tissue microenvironment, regular exercise improves lipolysis and fatty acid oxidation, which is perhaps required for the activation of M2 macrophages. Furthermore, with less metabolic stress, there would be a downregulation of pro-inflammatory signaling. A shift in pro-inflammatory signaling would also result in a reduced recruitment of circulating monocytes, which may also attenuate the polarization of M1 macrophages.

The beneficial effects of exercise, such as improved substrate utilization at rest, result in attenuation of metabolic stress in adipocytes, in part by upregulation of PPARγ-associated signaling. Improved metabolic efficiency in adipocytes may reduce the secretion of pro-inflammatory cytokines and attenuate the recruitment of monocytes, or restore the M2-polarization of macrophages (Figure 2). It is still unclear when the M2-to-M1 macrophage phenotype switch occurs during the onset of obesity, given that during both phenotypes are present in the obese adipose tissue, albeit at different time-points and in different proportions. It is also unclear how exercise training can result in a sustained form of one or both phenotypes in adipose tissue.

What remains unknown, in addition, is how other forms of exercise, namely resistance exercise, or non-load bearing exercise, such as swimming, may mediate the signaling pathways differently from that of the more typical endurance exercise. More studies are needed to investigate the underlying mechanisms that drive macrophage polarization in response to exercise and also delineate the M1-to-M2 phenotype shift after exercise training based on the consensus guidelines described in the Milan congress (14).

## Author Contributions

JG conceived, drafted, and prepared the manuscript. AA prepared the figures, and drafted part of the manuscript. KG critically appraised the manuscript and drafted part of the manuscript. All authors give permission for this manuscript to be published.

## Conflict of Interest Statement

The authors declare that the research was conducted in the absence of any commercial or financial relationships that could be construed as a potential conflict of interest.
